# Comparing Statistical Models to Predict Dengue Fever Notifications

**DOI:** 10.1155/2012/758674

**Published:** 2012-03-08

**Authors:** Arul Earnest, Say Beng Tan, Annelies Wilder-Smith, David Machin

**Affiliations:** ^1^Centre for Quantitative Medicine, Office of Clinical Sciences, Duke-NUS Graduate Medical School Singapore, Singapore 169857; ^2^Tan Tock Seng Hospital, Singapore 308433; ^3^Institute of Public Health, University of Heidelberg, Germany; ^4^National University of Singapore, Singapore 119077; ^5^University of Leicester, UK; ^6^University of Sheffield, UK

## Abstract

Dengue fever (DF) is a serious public health problem in many parts of the world, and, in the absence of a vaccine, disease surveillance and mosquito vector eradication are important in controlling the spread of the disease. DF is primarily transmitted by the female *Aedes aegypti* mosquito. We compared two statistical models that can be used in the surveillance and forecast of notifiable infectious diseases, namely, the Autoregressive Integrated Moving Average (ARIMA) model and the Knorr-Held two-component (K-H) model. The Mean Absolute Percentage Error (MAPE) was used to compare models. We developed the models using used data on DF notifications in Singapore from January 2001 till December 2006 and then validated the models with data from January 2007 till June 2008. The K-H model resulted in a slightly lower MAPE value of 17.21 as compared to the ARIMA model. We conclude that the models' performances are similar, but we found that the K-H model was relatively more difficult to fit in terms of the specification of the prior parameters and the relatively longer time taken to run the models.

## 1. Introduction

The incidence of dengue fever (DF) has grown dramatically around the world in recent decades, with some 2.5 billion people now at risk of the disease [1]. Dengue haemorrhagic fever (DHF) is a potentially lethal complication, with an estimated 500 000 people requiring hospitalization each year, a very large proportion of whom are children. About 2.5% of those affected die [1].

DF is a viral vector-borne disease that is common in the tropics and subtropics and is primarily spread by the female *Aedes aegypti* mosquito. Mosquito vector control is important in restricting its spread. It has been found that controlling the vector population before disease is detected reducing transmission with a reduction of the *Aedes aegypti *population in a 3-month period, from 16% to 2%, as measured by the premises index [2]. However, predicting the incidence of vector-borne diseases like DF remains difficult, as DF shows strong variations over time [3–5]. In Singapore, seasonal trends are seen with peaks occurring generally in June or September. DF is characterized by both epidemic peaks that appear every 3–5 years, as well as seasonal oscillations within a year. Possible reasons for changes in outbreak patterns include change in number of infections due to interventions to eradicate the mosquitoes, as well as change in the number of people who are susceptible to the disease through prior infections [6]. Seasonal trends in DF can be caused by several factors, including climatic variables such as temperature and precipitation [7–10].

Autoregressive Integrated Moving Average (ARIMA) models have been used in applications such as the assessment of seasonal variation in selected medical conditions [11], and as a surveillance tool for outbreak detection [12]. ARIMA (AR, D, MA) models make use of previous observations to make predictions of future values using lag parameter values. Lags of the differenced series appearing in the forecasting equation are termed Auto Regressive (AR), those of the forecast errors, Moving Average (MA), and a time series that needs to be differenced to achieve stationarity, Differenced (D). The prediction process uses constantly updated information (in our example weekly DF cases) to predict the course of dengue in subsequent weeks.

Time series analysis of infectious diseases within the Bayesian framework has been considered in some studies [13–16]. One such example demonstrated that *Klebsiella pneumoniae* is related to the quantity of a third-generation antibiotic use (cephalosporin) in a hospital, with a lag of three months [17]. Others included a Knorr-Held (K-H) two-component model to incorporate both seasonal and epidemic characteristics of notifiable infectious diseases [15], as well as a Bayesian hierarchical time series model to detect outbreaks of Rubella and Salmonella infections [14].

Studies have compared ARIMA models with dynamic models for infectious diseases (fitted via maximum likelihood methods) [18, 19]. However, to the best of our knowledge, a direct comparison between the single-component (ARIMA) and two-component (K-H) models has not been undertaken.

## 2. Methods

The purpose of this paper is to compare the two-component K-H with the single-component ARIMA model in predicting weekly DF notifications. Different formulations of models within each type are compared, together with a sensitivity analysis of the K-H model, fitted within a Bayesian framework.

### 2.1. Data

The Singapore Infectious Diseases Act (1977) requires medical practitioners to notify all cases of DF to the Ministry of Health (MoH) within 24 hours. We obtained data from the published “Weekly Infectious Disease Bulletin”, available from the MoH website which uses the World Health Organization 2009 criteria for DF which is also detailed there [20]. All notified and registered DF cases were laboratory confirmed, with laboratory assays from Polymerase Chain Reaction (PCR) and/or NS1 antigen (in the first 5 days of illness) and/or a positive Dengue Immunoglobulin M after day 5 of illness.

We studied weekly DF notifications in Singapore till June 2008. Data from January 2001 to December 2006 was used to estimate the model parameters. Thereafter, we performed external validation of the models using data from January 2007 to June 2008.

### 2.2. ARIMA Model

If *f*_*T*_ represents the number of cases of DF in week *T*, then AR relates this observation to an earlier *f*_*T*−*J*_, where *J* = 1,2,…, *T* − 1. MA relates the error (defined as the difference between the observed, *f*, and that predicted, *F*, notifications) at week *T* to week (*T* − *K*), where *K* = 1,2,…. D allows the differenced series, Δ*T* = (*f*_*T*_ − *f*_*T*−*L*_), to be modelled in the event of nonstationarity in the time series, where *L* = 0,1, 2,…. Here *J*, *K*, and *L* are the “orders” of the respective ARIMA components. Partial autocorrelation (PAC) and autocorrelation (AC) plots are used to determine *J* and *K*, respectively.

We describe the ARIMA (3,1,1) model equation used in our analysis. The number of cases of DF at week *T* is denoted as *f*_*T*_, where *T* is the first week for which DF is to be predicted

(1) $$F_{T}=\mu +f_{T-1}+\varphi_{1}f_{T-1}+\varphi_{2}f_{T-2}+\varphi_{3}f_{T-3}+\theta \varepsilon_{T-1}+\varepsilon_{T},$$

where *F*_*T*_ is the predicted number of DF cases for week *T*, and *f*_*T*−1_, *f*_*T*−2_, and *f*_*T*−3_ are the DF counts in the three immediate preceding weeks, termed lag 1, lag 2, and lag 3, respectively, and *ε*_*T*_, *ε*_*T*−1_ are the error term at time *T* and *T* − 1, respectively. In essence, we used observed values up till time *T* − 1 to predict for dengue fever cases at week *T*. *μ* is a constant and *φ*_1_, *φ*_2_, and *φ*_3_ are the coefficients for the three autoregressive terms in the model, *θ* is the first order moving average parameter and these are estimated within Stata V11.0 [21] via full or unconditional maximum likelihood estimates. For the ARIMA models, we used the Mean Absolute Percentage Error (MAPE) described below to compare predictive accuracy of the models.

### 2.3. Two-Component K-H Model

The K-H model distinguishes between the endemic, *x*, and epidemic, *y*, components of DF such that the number of cases observed *f*_*T*_ = *x*_*T*_ + *y*_*T*_ and the corresponding prediction model is formulated as

(2) $$F_{T}=X_{T}+Y_{T}.$$

Here *X*_*T*_ and *Y*_*T*_ have independent Poisson distributions with a composite parameters (*ω*_*T*_ *ν*_*T*_) and (*ω*_*T*_ *λ*_*T*_ *F*_*T*−1_), in which *ω*_*T*_ handles over dispersion, hence *F*_*T*_ is also Poisson with parameter *ω*_*T*_[*ν*_*T*_ + *λ*_*T*_ *F*_*T*−1_]. This in turn corresponds to a negative binomial distribution with dispersion parameter *ψ*. The mixing parameter, *ω*_*T*_, is assumed to have a Gamma distribution with parameters (*ψ* + *F*_*T*_) and (*ψ* + *ν*_*T*_ + *λ*_*T*_ *F*_*T*−1_).

The endemic parameter, *ν*_*T*_, is modelled as a harmonic wave (to handle strong seasonality inherent in infectious disease surveillance data) with

(3) $$\log\nu_{T}=\gamma_{0}+\gamma_{1}\sin\left(\frac{2\pi T}{52}\right)+\gamma_{2}\cos\left(\frac{2\pi T}{52}\right),$$

see [15], where 2*π*/52 is the base frequency of the curve, which is suitable for weekly data and *γ*_0_ is a constant. The logarithmic transformation is necessary to ensure stationarity in the variance of the series.

The epidemic component is derived from the parameter sequence ***λ*** = (*λ*_1_,…, *λ*_*n*_), which is assumed to be a piecewise constant [15] with unknown number of location *K* and unknown location of the changepoints *θ*_1_ < ⋯<*θ*_K_, that is,

(4) $$\lambda_{T}=\{\begin{array}{cc} \lambda^{\left(1\right)}, & \text{if}\;T=1,2,\ldots ,\theta_{1},\\ \lambda^{\left(k\right)}, & \text{if}\;T=\theta_{k-1}+1,\ldots ,\theta_{k},\\ \lambda^{\left(K+1\right)}, & \text{if}\;T=\;\theta_{K}+1,\ldots ,n, \end{array}$$

where *θ*_1_ < *θ*_2_ < ⋯<*θ*_*K*_ are the *K* unknown changepoints, such that *θ*_*k*_ ∈ {1,2,…, *n* − 1) for all *k* ∈ (1,2,…, *K*). For *K* = 0, there is no changepoint and *λ*_*T*_ = *λ* ^(1)^ for all *T* = 1,…, *n* [15]. The piecewise function is needed to provide flexibility in the model in terms of modelling the outbreaks of dengue fever in addition to possible seasonal trends that we observe.

The two-component model formulation is completed by specifying prior distributions for the parameters in the model as follows:

(5) $$\begin{array}{c} \gamma \sim N\left(0,\sigma_{\gamma }^{2}I\right),\\ \lambda \sim \text{Ga}\left(\chi_{\xi },\delta_{\xi }\right),\\ \psi \sim \text{S}\left(\alpha_{\psi },\beta_{\psi }\right) \end{array}$$

*N* denotes a normal and Ga a Gamma distribution. *σ*_*γ*_^2^ was set to 10^6^, representing highly dispersed independent normal priors for each coefficient. **I** is an identity matrix. For *λ* ^(*k*)^, *k* = 1,…, *K* + 1, independent exponential distributions with mean 1/*ξ* and variance 1/*ξ* ^2^ were specified. *ξ* was then assigned a gamma hyperprior Ga(*χ*_*ξ*_, *δ*_*ξ*_). The marginal prior distribution for *λ* ^(*k*)^ is then a gamma-gamma distribution [22]. In our study, we used *χ*_*ξ*_ = 10 and *δ*_*ξ*_ = 10, which corresponded to the gamma-gamma marginal of *λ* ^(*k*)^ turning out to be an *F*-distribution with (2,20) degrees of freedom, which then indicates that the marginal prior probability of an outbreak occurring (i.e., *λ* ^(*k*)^ ≥ 1) is 0.39, while always favouring smaller values of *λ* ^(*k*)^, with the density function monotonically decreasing. The dispersion parameter for the negative binomial distribution, *ψ*, which was designed to handle extra-Poisson variation in the data, was assigned a gamma hyperprior as well, with the following parameter, Ga(*α*_*ψ*_, *β*_*ψ*_).  *α*_*ψ*_ and *β*_*ψ*_ were assigned values of 1 and 0.1, respectively in the original analysis corresponding to a prior mean and standard deviation of 10.

The K-H models were fitted using the customised Bayesian software Twins V1.0 [15]. Markov Chain Monte Carlo (MCMC) methods, in particular the Metropolis-Hastings algorithm, were used to estimate the parameters. For each model, we ran 200 iterations as burn-ins. These burn-in samples were discarded and not used in the analysis. We ran a further 60,000 iterations, but only saved every 20th observation, resulting in a final 3000 sample size. This was to circumvent the problem of autocorrelated samples.

### 2.4. Model Comparison

We compared the ARIMA model with the K-H model and as well conducted a sensitivity analysis on the K-H model using the MAPE:

(6) $$\text{MAPE}=\frac{1}{n}\overset{n}{\underset{T=1}{\sum }}\left|\frac{f_{T}-F_{T}}{F_{T}}\right|,$$

where *n* is the total number of weeks of data. 

The Bayesian analyses were based on several assumptions regarding the prior distributions, and we assessed the robustness of our results in a sensitivity analysis. For the sensitivity analyses, we considered 4 different scenarios which involved varying values of *χ*_*ξ*_, *δ*_*ξ*_ or *α*_*ψ*_, *β*_*ψ*_ while keeping the other variables at their original values: Model 1: *α*_*ψ*_ = 0.1 and *β*_*ψ*_ = 0.1, Model 2: *α*_*ψ*_ = 10 and *β*_*ψ*_ = 1, Model 3: *χ*_*ξ*_ = 1 and *δ*_*ξ*_ = 1, and Model 4: *χ*_*ξ*_ = 10 and *δ*_*ξ*_ = 1. The prior values for the sensitivity analysis were selected to represent a range of realistic scenarios where the probabilities of an outbreak were expected to be different. In particular, we selected priors where the probability of observing an outbreak ranged from 0.001 (for *χ*_*ξ*_ = 10 and *δ*_*ξ*_ = 1) to 0.5 (*χ*_*ξ*_ = 1 and *δ*_*ξ*_ = 1).

## 3. Results

**Figure 1:**
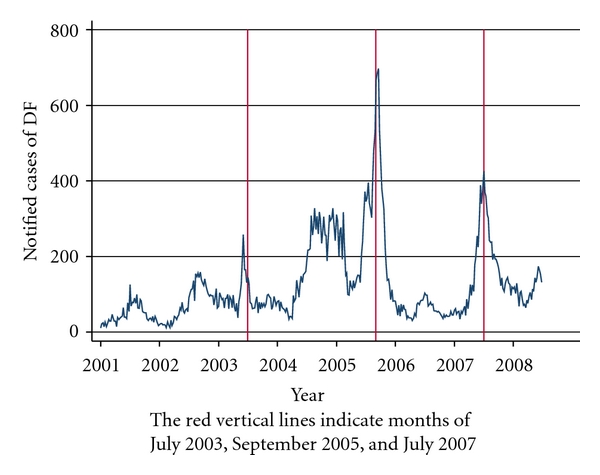
Weekly cases of dengue fever (DF) in Singapore.

Figure 1 highlights the weekly distribution of DF notifications in Singapore from January 2001 to June 2008. It is evident that DF notification exhibits both seasonal trends (e.g., regular peaks around June or September and troughs seen in the first 4 months of the year) and epidemic trends (most markedly shown during the 2005 epidemic, when average weekly counts exceeded 600 cases).

**Figure 2:**
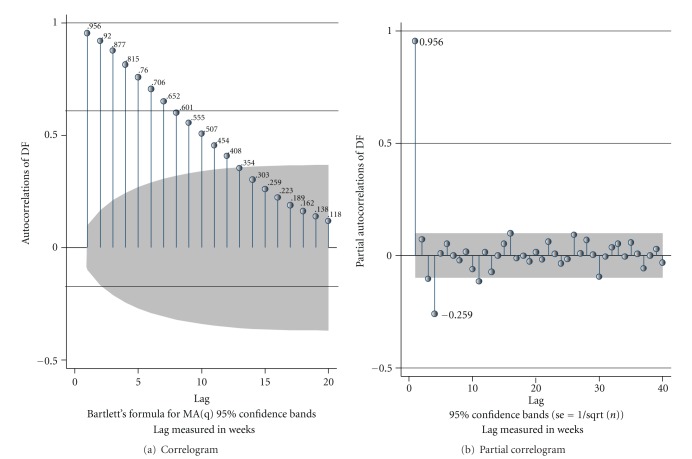
Plots of autocorrelation and partial correlation for dengue fever (DF).

The autocorrelation plots for DF (Figure 2(a)) indicated that correlations gradually declined over the weeks to insignificant values after 12 weeks. The partial autocorrelations plots (Figure 2(b)) showed a spike at week 1 and week 4 indicating possible inclusion of AR terms of the order of up to four in the ARIMA model. We evaluated the various combinations, including autocorrelation terms 3 and 4 in our analysis.

We explored various formulations of the ARIMA model, and we summarise some of the more important ones in Table 1. As can be seen, ARIMA (3,1,0) provided the lowest MAPE value of 19.86. Including a moving average term did not improve the fit of the model, as with adding an autocorrelation term of four. Adding a 12-month seasonal component (not shown) also did not lower the MAPE. The parameters for the final ARIMA model are shown in Table 2. We found all three autoregressive terms AR(1) = −0.10 (*P* = 0.001), AR(2) = 0.10 (*P* = 0.002), and AR(3) = 0.23 (*P* < 0.001) to be statistically significant. The parameters for the K-H model are also provided in Table 2.

**Figure 3:**
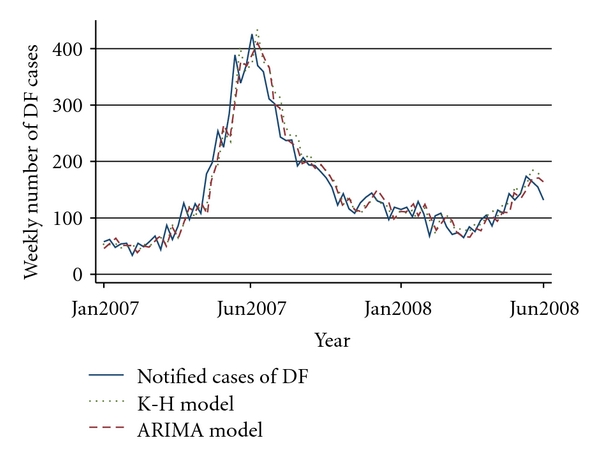
Comparison of out-of-sample forecasts of dengue fever (DF) between ARIMA and two-component K-H model (January 2007 to June 2008).

The comparison between the ARIMA and K-H model is shown in Figure 3.  Table 3 shows the results from comparing the two models. Overall, the K-H model performed marginally better than the ARIMA model (MAPE of 17.21 and 17.54 resp.). In particular, the model predicted well (out-of-sample) for certain periods, including the early endemic periods between weeks 1 to 12. Fine-tuning the parameters for the K-H model allowed us to make better predictions for the epidemic periods, as we show in the sensitivity analysis (Table 4). For instance, the model predicted well for the epidemic periods within the weeks 17 to 24 (sensitivity analysis 4).

In terms of forecasting one-week ahead DF notifications, both methods performed well (Figure 3). For instance, the K-H model forecasted 53 (observed 58) for 2007 week 1, 356 (observed 371) for 2007 week 26, 112 (observed 115) for 2008 week 1, and 171 (observed 132) for 2008 week 26. It is worth noting that these results are for out-of-sample predictions.

The Bayesian analysis is influenced by the prior specification. As such, we investigated the robustness of our results to different formulation of the priors. These priors represented a wide range of realistic scenarios where the probability of an outbreak is expected to differ. As can be seen from Table 4, it appears that the models have generally similar MAPE values except for sensitivity analysis 4, where the MAPE is actually the lowest at 16.54. In our local setting, we found that specifying a small prior probability of 0.001 for an outbreak to occur provided a better fit of the data.

## 4. Discussion

We found that the K-H model performed better than the conventional ARIMA time series model; however, this was only marginal. Forecasting weekly cases of DF has immense implication for hospital resources planning. For an infectious disease ward, knowing the normal trend of DF, along with predictions of the following week's DF can allow hospital planners to better plan for and allocate their manpower and other resources. Intensive media campaigns (e.g., television advertisements) in the weeks prior to a projected increase in DF notifications may prove to reduce the number of new cases.

Though we used the MAPE index to compare the models, other indices are also available. The Mean Squared Error, for instance, is calculated from the sum of the squared error values. Compared to MAPE, the values are not relative to the magnitude of the observation, and the values are not intuitively easy to interpret.

There were several limitations in our study. Firstly, our analysis was dependent on notifiable data. While clinicians are required to report all cases of DF and DHF to the MOH, there is a possibility that the cases could be underreported, especially since mild asymptomatic cases of DF may have not been diagnosed. While this may have led to an under-estimate in the forecasts, the comparisons across the models are still valid, as they make use of the same number of weekly cases.

In our analysis, we compared the predictive capability of the models using one-week ahead forecast of dengue fever notification. It is possible to forecast for periods longer than that, of course the predictions may inherently not be as accurate as a one-week forecast.

In conclusion, we found that both the final models chosen for the ARIMA and K-H models predict the future course of DF in Singapore reliably well, while the former performed marginally better. The ARIMA models were relatively faster to implement and run, while the K-H model was sensitive to the choice of priors, which needs to be carefully made before the study is conducted.

## Figures and Tables

**Table 1:** Comparison of MAPE values across various ARIMA models.

Model	Model specification	MAPE
1	ARIMA (1,0,0)	23.61
2	ARIMA (2,0,0)	23.09
3	ARIMA (3,0,0)	23.20
4	ARIMA (4,0,0)	23.23
5	ARIMA (3,1,0)	**19.86**
6	ARIMA (3,1,1)	19.96

**Table 2:** Parameters for the final models.

ARIMA model	Coefficient	95% confidence interval		*P* value
Constant (*μ*)	0.28	−3.86	4.41	0.896
AR 1 (*φ*_1_)	−0.10	−0.16	−0.04	0.001
AR 2 (*φ*_2_)	0.10	0.04	0.17	0.002
AR 3 (*φ*_3_)	0.23	0.17	0.29	<0.001

K-H model	Coefficient	95% credible interval		

*ψ*	25.1	18.4	32.3	
*γ*_0_	3.3	1.9	3.6	
*γ*_1_	−0.2	−0.3	0.3	
*γ*_2_	−0.5	−0.7	−0.4	
*ξ*	1.0	1.0	1.0	
*K*	7.6	0.01	15.0	
*λ*	1.3	0.7	2.0	

**Table 3:** Comparison of out-of-sample predictions (external validation) between ARIMA and K-H models.

MAPE	ARIMA	K-H
Overall	17.54	**17.21**

Stratified (in 4 week intervals)		
*Year 2007*		
Weeks 1 to 4	17.07	**14.27**
5 to 8	28.60	**25.62**
9 to 12	33.41	**30.63**
13 to 16	32.52	33.09
17 to 20	21.83	**20.53**
21 to 24	20.64	**19.76**
25 to 28	12.86	13.22
29 to 32	11.53	14.40
33 to 36	8.54	10.26
37 to 40	5.07	6.50
41 to 44	18.49	**17.42**
45 to 48	8.54	10.35
49 to 52	15.70	**12.44**
*Year 2008*		
Weeks 1 to 4	11.13	11.16
5 to 8	29.09	**25.63**
9 to 12	16.39	19.41
13 to 16	15.51	**10.77**
17 to 20	19.21	**18.38**
21 to 24	9.83	10.07

**Table 4:** Sensitivity analysis on K-H model parameters.

MAPE	Initial K-H model	Sensitivity analysis			
		1	2	3	4
Overall	17.21	17.71	17.71	17.50	16.54
Stratified (in 4 week intervals)					
*Year 2007*					
Weeks 1 to 4	14.27	20.12	22.33	20.30	20.03
5 to 8	25.62	25.41	25.66	25.12	23.39
9 to 12	30.63	31.06	31.30	30.95	31.07
13 to 16	33.09	33.20	32.31	32.49	27.89
17 to 20	20.53	20.41	20.40	20.92	21.82
21 to 24	19.76	21.14	20.90	21.25	21.58
25 to 28	13.22	13.45	14.18	13.28	12.91
29 to 32	14.40	13.98	13.04	13.39	10.65
33 to 36	10.26	10.76	9.97	10.55	6.11
37 to 40	6.50	6.69	6.39	5.54	3.30
41 to 44	17.42	17.62	17.54	16.91	15.94
45 to 48	10.35	11.30	10.59	11.09	10.67
49 to 52	12.44	12.99	12.37	12.58	13.12
*Year 2008*					
Weeks 1 to 4	11.16	11.09	10.85	11.13	11.31
5 to 8	25.63	25.53	26.01	25.49	25.83
9 to 12	19.41	20.03	20.25	19.31	16.28
13 to 16	10.77	10.72	10.47	10.72	11.25
17 to 20	18.38	19.20	17.97	18.59	19.27
21 to 24	10.07	9.33	10.98	9.95	9.92

A description of the parameters used in the sensitivity analysis is provided in the 4th page of the manuscript.
